# Calcium signaling in astrocytes and gliotransmitter release

**DOI:** 10.3389/fnsyn.2023.1138577

**Published:** 2023-03-02

**Authors:** Julianna Goenaga, Alfonso Araque, Paulo Kofuji, Daniela Herrera Moro Chao

**Affiliations:** Department of Neuroscience, University of Minnesota, Minneapolis, MN, United States

**Keywords:** astrocyte, gliotransmission, tripartite synapse, plasticity, calcium signaling

## Abstract

Glia are as numerous in the brain as neurons and widely known to serve supportive roles such as structural scaffolding, extracellular ionic and neurotransmitter homeostasis, and metabolic support. However, over the past two decades, several lines of evidence indicate that astrocytes, which are a type of glia, play active roles in neural information processing. Astrocytes, although not electrically active, can exhibit a form of excitability by dynamic changes in intracellular calcium levels. They sense synaptic activity and release neuroactive substances, named gliotransmitters, that modulate neuronal activity and synaptic transmission in several brain areas, thus impacting animal behavior. This “dialogue” between astrocytes and neurons is embodied in the concept of the tripartite synapse that includes astrocytes as integral elements of synaptic function. Here, we review the recent work and discuss how astrocytes *via* calcium-mediated excitability modulate synaptic information processing at various spatial and time scales.

## Introduction

Nervous systems throughout the animal kingdom vary in structure and complexity and are made up of neurons, specialized cells that can receive and transmit chemical or electrical signals, and glial cells, historically considered to only provide support functions to neurons. Glial cells were first described by Virchow in the 1850s as “nervenkitt” or nerve glue, implying a homogenous population of support cells holding them together (García-Marín et al., 2007). However, several different types of glia can be differentiated based on their different functions and morphology. Among them, there are microglia, oligodendrocytes, and astrocytes. The term astrocyte was coined by Michael von Lenhossek to describe star-shaped cells observed in histological brain specimens (Parpura and Verkhratsky, 2012). Subsequently, Camillo Golgi and Ramon y Cajal with the development of novel histological stains illustrated several astrocytes with their elaborated processes (García-Marín et al., 2007; Navarrete and Araque, 2014). Conventionally, two major classes of astrocytes have been distinguished in histological sections of the central nervous system (CNS) based on their morphology and distribution, the fibrous and protoplasmic astrocytes (Miller and Raff, 1984). The fibrous astrocytes are located mainly in white matter with few straight and long processes. Their processes are long (up to 300 μm), though much less elaborate as compared to protoplasmic astroglia. The protoplasmic astrocytes are mainly found in gray matter and are characterized by their extremely elaborate morphology with many branching processes yielding a “bushy” or “spongiform” appearance. Protoplasmic astrocytes extend their endfeet to blood vessels and enwrap them to form the glial limiting membrane, which is the outermost wall of the blood–brain barrier (BBB). More recently, the emergence of molecular approaches such as RNA-sequencing and proteomic analysis has revealed a much larger degree of astrocytic heterogeneity across various brain regions. Excellent reviews related to this topic can be found elsewhere (Zhang and Barres, 2010; Farmer and Murai, 2017; Miller, 2018; Xin and Bonci, 2018; Matias et al., 2019).

Astrocytes customarily have been identified using the intermediary filament protein Glial Fibrillary Acid Protein (GFAP) as a histological marker (Shehab et al., 1990; Zhang et al., 2019; Batiuk et al., 2020; Jurga et al., 2021). Other markers such as the enzyme glutamine synthetase or a Ca^2+^ binding peptide S100 have also been applied (Norenberg, 1979; Gonçalves et al., 2008). Transcriptome analysis of purified astrocytes identified novel molecular markers for astrocytes such as aldehyde dehydrogenase family 1 member L1 (Cahoy et al., 2008) or the transcription factor Sox9 (Sun et al., 2017).

Electrophysiologically, astrocytes are characterized by their lack of voltage-gated conductances, displaying a quasi-linear voltage-current relationship (Stevens and Wang, 1995). The expression of large amounts of inwardly rectifying potassium channels confers astrocytes with their characteristic low input resistance and membrane potential close to the equilibrium potential for transmembrane potassium. The principal potassium channels are the weakly inwardly rectifying Kir4.1 channels (Nwaobi et al., 2016) although other potassium channels such as the two-pore domain TWIK-1 and TREK-1 channels are also likely to be expressed in astrocytes (Zhou et al., 2009). Another major conductance found in astrocytes is the connexin channel such as connexin 43 which provides gap junctional coupling among astrocytes (Nagy and Rash, 2000). This gap junctional coupling allows the intercellular passive diffusion of endogenous signaling molecules, such as inositol (1,4,5)-triphosphate (IP3) (Leybaert et al., 1998), as well as glucose and its metabolites, glutamate, glutamine, and lactate (Medina et al., 1999). Therefore, astrocytes are considered to form a functional network of communicating cells.

Astrocytes also express various transporter proteins on the plasma membrane for the uptake of neurotransmitters. Transporters are vital for the normal CNS physiology by maintaining neurotransmitter homeostasis and modulating synaptic transmission. It is estimated that astrocytes remove about 80% of the glutamate released, whereas the remaining 20% is taken up by neurons (Parpura and Verkhratsky, 2012). Astrocytes remove extracellular glutamate by excitatory amino acid transporters (EAAT). Five types of EAATs are present in the human brain; the EAAT1 and EAAT2 are expressed almost exclusively in astrocytes (the rodent analogs are known as glutamate/aspartate transporter, GLAST, and glutamate transporter-1, GLT-1) (Murphy-Royal et al., 2017; Mahmoud et al., 2019).

Studies in the past few years have shown that astrocytes are spatially organized to form exquisite tridimensional structures (Gavrilov et al., 2018; Refaeli et al., 2021; Aten et al., 2022). Reconstruction of protoplasmic astrocyte assemblies in the rat hippocampus showed that astrocyte cell bodies are evenly spaced, and their processes overlap only minimally creating a “tiling” of astrocytes (Bushong et al., 2002; Ogata and Kosaka, 2002). This may be the case in some other brain regions as well (Halassa et al., 2007) though overlap of astrocyte territories have also been described (López-Hidalgo et al., 2016). Perhaps even more surprising is how a single astrocyte with its large territory and complex morphology can massively interact with a neuronal network. Indeed, a single astrocyte in the rat hippocampus is estimated to occupy a territory of 66,000 μm^3^ of neuropil and contact over 140,000 synapses (Bushong et al., 2002).

**FIGURE 1:**
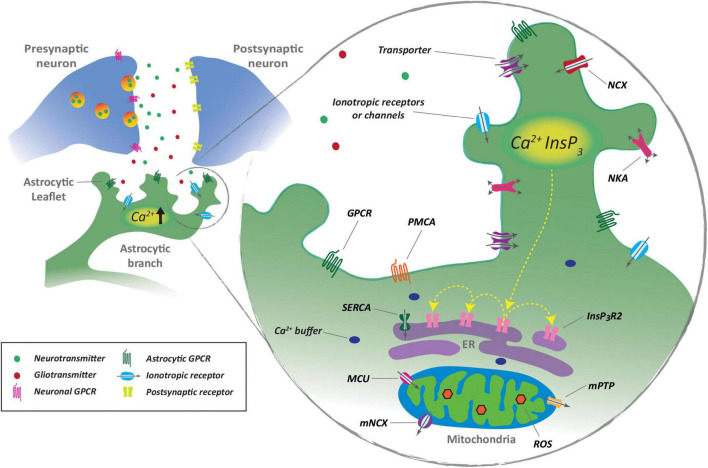
Synaptic regulation of astrocyte Ca^2+^ signaling. Astrocyte leaflets sense and respond to synaptic activity through neurotransmitter receptors and transmitter transporters. Ca^2+^ transients are triggered by Ca^2+^ entry and by Ca^2+^ release from the endoplasmic reticulum (ER) through inositol 1,4,5-triphosphate receptors (IP3R) after G-protein-coupled receptor (GPCR) activation. Mitochondria also participate in Ca^2+^ loci by action of mitochondrial permeability transition pore (mPTP) and mitochondria sodium/calcium exchanger (NCX). Ca^2+^ can be removed from the cell by action of Ca^2+^ ATPase (PCMA) or mobilized to the ER by sarcoplasmic/endoplasmic reticulum calcium ATPase (SERCA). NKA, Na+/K+ ATPase; InsP_3_, inositol 1,4,5-trisphosphate; MCU, mitochondria Ca^2+^ uniporter; ROS, reactive oxygen species.

As discussed above, the role of astrocytes in promoting neurotransmitter clearance at synapses has long been recognized. A more unconventional role of astrocytes at synapses has emerged in the last three decades. The deployment of calcium imaging techniques in cultured cells and in brain slices provided evidence that when neurons communicate with each other they also signal to astrocytes. In turn, astrocytes respond to this neuronal signaling by releasing various neuroactive substances, mentioned in detailed in the section below, such as ATP, glutamate, D-serine, and GABA. Thus, the astrocytes form the third element at the synapses. Not only the information flows from presynaptic to postsynaptic elements but also streams to astrocytes that, in turn, regulate synaptic communication. This intimate morphological and functional association of astroglial processes in a synapse led to the conceptual term of a “tripartite synapse” (Figure 1).

## Astrocytic Ca^2+^ excitability

The plasticity of neuronal connectivity requires dynamic cooperation between neurons and astrocytes (Allen and Eroglu, 2017). Astrocytes change their morphology and synaptic coverage to scale synaptic strength and modulate neuronal circuit activity (Gómez-Gonzalo et al., 2017; Verkhratsky and Nedergaard, 2018; Henneberger et al., 2020; Semyanov and Verkhratsky, 2021). Although not electrically excitable, astrocytes display complex intracellular Ca^2+^ pathways as a major component of astrocytic signaling. Interaction between synapses and astrocytic arborization promotes astrocyte Ca^2+^ events to modulate astrocyte neurotransmitter and K^+^ uptake, the release of neuroactive molecules (Wang et al., 2012; Zorec et al., 2012; Araque et al., 2014), and regulation of local blood flow (Petzold and Murthy, 2011; MacVicar and Newman, 2015). Astrocyte Ca^2+^ events manifest differentially in space and time within single astrocytes and across astrocytic networks (Semyanov et al., 2020). In soma and primary branches, Ca^2+^ events are primarily initiated by intracellular Ca^2+^ release from Ca^2+^ stores in the endoplasmic reticulum (ER) and mitochondria (Verkhratsky et al., 2018). Moreover, astrocytic Ca^2+^ transients mainly have also been suggested to occur by Ca^2+^ entry through the plasma membrane following Na^+^ increases during neurotransmitter uptake *via* the sodium/calcium exchanger (NCX) (Verkhratsky et al., 2018) or after activation of other ionotropic Ca^2+^ permeable receptors and transient receptor potential channels (Shigetomi et al., 2011, 2013; Shibasaki et al., 2014; Rakers and Petzold, 2017).

In contrast to neurons, astrocytes contain processes with distinct morphology and complement organelles that generate widely distributed Ca^2+^ loci that allow them to differentially respond to synaptic activity and integrate multiple synaptic inputs (Perea and Araque, 2005; Bernardinelli et al., 2014; Semyanov et al., 2020). Astrocytic branches are intermingled with neuronal structures and contain Ca^2+^ stores that can trigger and amplify Ca^2+^ events by activation of inositol-1,4,5-triphosphate receptors (IP3Rs). IP3Rs are synergistically modulated by IP3 and Ca^2+^ levels and further Ca^2+^-dependent phospholipase C activation, stimulating Ca^2+^ release from the ER (Foskett et al., 2007; Khakh and Sofroniew, 2015). Ca^2+^ levels can also reach the threshold for activation of IP3Rs by activation of plasmalemmal G-protein-coupled receptors (GPCRs) (Semyanov and Verkhratsky, 2021) and *via* increased diffusion of Ca^2+^ from multiple daughter leaflets (Semyanov, 2019). Intracellular Ca^2+^ amplification between clusters of IP3Rs can propagate Ca^2+^ waves within the astrocyte cell body and further astrocytic branches (Srinivasan et al., 2015; Semyanov and Verkhratsky, 2021). Ca^2+^ event generation in leaflets can be additionally enhanced by ER-independent release mechanisms, involving Ca^2+^ efflux from mitochondria, in response to the transient opening of permeability transition pores (Agarwal et al., 2017; Figure 1).

The analysis of the neurotransmitter-evoked astrocyte calcium dynamics has revealed that astrocytes integrate incoming synaptic information (Perea and Araque, 2005; Shigetomi et al., 2008). Indeed, synaptic action of excitatory or inhibitory neurotransmitters evoke non-linear calcium elevations and result in the control of the spatial propagation of the intracellular calcium signal within the astrocyte (Perea and Araque, 2005; Shigetomi et al., 2008; Mariotti et al., 2016; Durkee and Araque, 2019; Liu et al., 2022), which is indicative of synaptic information processing by astrocytes. The control of the spatial extent of the calcium signal may have important functional consequences, as it may regulate the spatial extention of the gliotransmitter release and the consequent synaptic regulation (Durkee and Araque, 2019). Moreover, converging Ca^2+^ signals from multiple daughter leaflets can be finally integrated by parent branches as a readout of local network activity (Lock et al., 2019). In some circumstances, propagating Ca^2+^ waves can spread through astrocytes and the astrocytic network to influence neuronal activity. This pathway has been suggested to guide information processing across neuronal networks (Tong et al., 2013; Semyanov and Verkhratsky, 2021). Ca^2+^ events are terminated by Ca^2+^ removal through the plasma membrane by Ca^2+^ ATPase (PCMA) or by uptake to Ca^2+^ stores by ER calcium ATPase (SERCA) (Bazargani and Attwell, 2016). Elongated mitochondria in astrocytic branchlets can also actively uptake intracellular Ca^2+^ by mitochondria Ca^2+^ uniporters (Zhang and Ding, 2018).

## Heterogeneity of astrocytic Ca^2+^ signals

Astrocytic Ca^2+^ events can be classified as either spontaneous or neurotransmitter-evoked (Khakh and McCarthy, 2015; Semyanov et al., 2020). Spontaneous events are characterized by intrinsic Ca^2+^ fluctuations that can occur in the absence of external signals (Nett et al., 2002; Wang et al., 2006). These spontaneous Ca^2+^ oscillations persist even if neuronal firing or neuronal and astrocytic vesicular release is blocked (Wang et al., 2006; Sun et al., 2014). Even though the precise mechanisms mediating the triggering of spontaneous Ca^2+^ transients are not completely understood, it has been proposed that they can be the result of stochastic Ca^2+^ fluxes through simultaneous multiple pathways (Ding et al., 2018; Denizot et al., 2019). These mechanisms involve both entering Ca^2+^ from the extracellular space through Ca^2+^ permeable receptors, Ca^2+^ channels, and Na^+^/Ca^2+^ exchangers at the plasma membrane or intracellular Ca^2+^ stores through IP3Rs on the ER and mitochondrial permeability *via* transition pores (Rungta et al., 2016; Agarwal et al., 2017; Wu et al., 2019). The addition of small spatially determined Ca^2+^ events stimulates local cytosolic Ca^2+^ oscillations that can trigger Ca^2+^-dependent Ca^2+^ release *via* activation of IP3Rs, leading to amplification and propagation of Ca^2+^ events (Khakh and McCarthy, 2015). The magnitude of spontaneous Ca^2+^ activity can be influenced by the intrinsic activity of Gq GPCRs, which stimulates sufficient levels of IP3 to activate IP3Rs, or by focal points of elevated Ca^2+^ which acts as a co-agonist of IP3Rs. Ca^2+^ fluxes can be further strengthened or weakened depending on cellular energy states, changes in membrane potential, surface-to-volume ratio, and ER depletion (Khakh and McCarthy, 2015; Ding et al., 2018; Stobart et al., 2018). In soma and primary branches, intracellular Ca^2+^ waves will mobilize in a specific spatial path within the cell, depending on the proximity of ER IP3Rs, further distance from IP3Rs will terminate the cascade and buffer Ca^2+^ to basal levels (Denizot et al., 2019).

### Astrocytic calcium signals in the soma and processes

Astrocytic Ca^2+^ signals are considered to rely mainly on the IP3R pathway, especially in the soma and primary branches, as genetic deletion of IP3R2, which is known to be enriched in astrocytes, reduces spontaneous Ca^2+^ oscillations with the complete abolition of Ca^2+^ signals in astrocytic soma. Residual Ca^2+^ activity in astrocyte processes, even if reduced, is still persistent in astrocytes of IP3R2^–/–^ mice (Kanemaru et al., 2014), suggesting IP3R-independent Ca^2+^ release mechanisms, especially in processes (Patrushev et al., 2013). Such mechanisms involve low cytosolic Ca^2+^ elevations in mitochondria (Agarwal et al., 2017; Okubo et al., 2019) and transmembrane Ca^2+^ fluxes mediated by transient receptor potential ion channels (TRPA1), that contribute to the maintenance of basal Ca^2+^ levels within astrocytes (Shigetomi et al., 2010, 2011). Importantly, 80% of the astrocyte Ca^2+^ activity *in vivo* takes place in astrocytic ramifications, that account for 75% of astrocytic volume (Bindocci et al., 2017). Spatial restriction of spontaneous Ca^2+^ events has been reported in *ex vivo* and *in vivo* preparations. Such events occur predominantly in distal parts of astrocyte processes and do not propagate to the soma, thereby identifying autonomous functional domains called “microdomains” (Grosche et al., 1999; Lia et al., 2021). High-resolution imaging techniques have allowed a deeper understanding of the distinct properties and mechanisms underlying astrocyte somatic and microdomain Ca^2+^ activity. While somatic Ca^2+^ increases can be triggered by intense neuronal firing patterns, astrocytic processes also respond to local levels of synaptic activity, suggesting compartmentalized astrocyte neuronal communication integration. Microdomain Ca^2+^ oscillations are more frequently observed than somatic ones and occur asynchronously in various processes (Volterra et al., 2014). Microdomain Ca^2+^ events have been deferentially categorized based on their distinct properties, however, a rich diversity of Ca^2+^ signals are present within single astrocytes and are modulated by local brain environments in distinct brain areas (Shigetomi et al., 2013; Khakh and Sofroniew, 2015). Previous elegant classifications have distinguished microdomain Ca^2+^ activity in focal and expanded microdomains (Di Castro et al., 2011; Clarke and Barres, 2013). Focal microdomains, also later referred to in the field, as localized microdomains in branches and branchlets (Khakh and Sofroniew, 2015), depend largely on IP3R-dependent Ca^2+^ transients and seem to be independent of neuronal firing. A distinct hypothesis has suggested that these events could originate from spontaneous neurotransmitter release at neighboring synapses, potentially contributing to plastic adaptations at the tripartite synapse (Di Castro et al., 2011; Clarke and Barres, 2013).

On the other hand, expanded microdomains present different Ca^2+^ dynamics, compared to focal events, with larger amplitude, duration, and spatial extent, and are highly sensitive to surrounding neuronal firing. The increased magnitude of these Ca^2+^ events has been suggested to result from the synchronization of several autonomous microdomains and might represent a more coordinated Ca^2+^ response that could modulate gliotransmitter release probability (Di Castro et al., 2011; Panatier et al., 2011; Volterra et al., 2014).

### Astrocytic calcium signaling in response to neuronal activity

Astrocytes sense, react and modify the extracellular transmitter homeostasis by responding *in situ* to neuronal activity. *Ex vivo* and *in vivo* examinations have provided strong evidence showing that neuronal inputs trigger astrocyte Ca^2+^ events by activation of multiple plasma membrane receptors (Nimmerjahn et al., 2004; Wang et al., 2006; Caudal et al., 2020; Figure 1). Engagement of distinct receptor arrays after neuronal input increases cytosolic IP3 levels and IP3R activation, promoting Ca^2+^ release from ER Ca^2+^ stores (Bazargani and Attwell, 2016). Additional Ca^2+^ entry to the cytosol and further triggering of Ca^2+^ transients can be observed after neuronal-mediated activation of ionotropic receptors, such as glutamate AMPA and NMDA (Saab et al., 2012), purinergic P2X (Abbracchio and Verderio, 2006), and nicotinic cholinergic receptors (Aryal et al., 2021) or after uptake of glutamate and GABA *via* Na^+^ influx *via* Na^+^/Ca^2+^ exchangers (Boddum et al., 2016; Brazhe et al., 2018; Rose et al., 2020). Evidence collected through the last decades has shown that astrocyte GPCR activation mainly leads to intracellular Ca^2+^ increases (Kofuji and Araque, 2021). Such a dynamic seems to oppose canonical responses observed in neuronal activation, as increases in astrocytic intracellular Ca^2+^ are triggered after activation of excitatory or inhibitory transmitter receptors (Mariotti et al., 2016; Perea et al., 2016) or other Gq, Gs, or Gi-coupled metabotropic receptors (Durkee and Araque, 2019; Yu et al., 2020). The consequences of GPCR-mediated increase in astrocytic Ca^2+^ are not fully characterized, however, exciting evidence has suggested that astrocytes can discriminate and integrate metabotropic signaling upstream of internal Ca^2+^ oscillations (Caudal et al., 2020). Different activation efficiencies of GPCRs exert equivalent (Shigetomi et al., 2008) or do not necessarily induce the release of gliotransmitters, contrary to the effects observed after Ca^2+^ uncaging or IP3 application (Wang et al., 2013).

Neuronal influence on astrocytic activity can occur at individual synapses but also after diffusion of neuromodulators, such as dopamine, acetylcholine, serotonin, and noradrenaline, that modulate spatiotemporal spontaneous Ca^2+^ events that trigger new Ca^2+^ fluctuations (Takata et al., 2011; Ding et al., 2013; Jennings et al., 2017; Corkrum et al., 2020; Semyanov et al., 2020). Evidence collected during the last decades has suggested that the modulation of astrocyte intracellular Ca^2+^-induced by neuromodulators finely tunes K^+^ homeostasis and gliotransmitter release (Wang et al., 2012; Pacholko et al., 2020). By integrating the neuromodulatory effects, astrocytes act as crucial players in behavioral states. Neuromodulator effects have been especially evident in astrocyte Ca^2+^ network activity, as they influence astrocyte activity thresholds in response to local neuronal activity or depending on the brain’s vigilance state (Ding et al., 2013; Araque et al., 2014). Astrocyte Ca^2+^ events in leaflets and branchlets can also be triggered by gliotransmitters or other diffuse signals in the local environment, as well as by changes in partial pressures of CO_2_ and O_2_, osmotic pressure, pH, and temperature (Angelova et al., 2015; Turovsky et al., 2016; Kofuji and Araque, 2021; Semyanov and Verkhratsky, 2021). Astrocytic Ca^2+^ activity resulting from the interaction between astrocytic processes and synapses can trigger astrocyte morphological remodeling and gliotransmitter release, which feedback to neuronal network excitability and functioning (Kofuji and Araque, 2021).

### Kinetics of astrocyte Ca^2+^ signals

Astrocyte Ca^2+^ signals in response to external stimulation present different temporal and spatial properties than neuronal activity. The timescale of astrocytic Ca^2+^ dynamics is generally much slower, with variable intervals between sensory stimulation and the onset of astrocytic Ca^2+^ event. Single action potentials that can last within a range of a few milliseconds differentiate from astrocytic Ca^2+^ events, as they can occur over durations of several hundred milliseconds to a few seconds (Paukert et al., 2014; Otsu et al., 2015). The differences in Ca^2+^ dynamics between neurons and astrocytes have raised the question of whether the astrocytic activity can be directly correlated to real-time information processing in the brain (Semyanov, 2019; Semyanov et al., 2020). Astrocyte information processing could potentially bridge information received by thousands of synapses belonging to different circuits and neurons and integrate the information in different spatial-temporal scales (Bushong et al., 2002; Perea and Araque, 2005; Halassa et al., 2007; Gordon et al., 2008). Indeed, recent evidence suggests that astrocytes could encode information by evoking specific time and spatial Ca^2+^ signal patterns, characterized by the different total area of appearance, number, and duration of Ca^2+^ events (Perea and Araque, 2005; Volterra et al., 2014; Nakayama et al., 2016; Wang et al., 2019).

Moreover, during information processing, astrocytes could incorporate not only, neuronal information, but also signals resulting from complex interactions with other non-neuronal cells and non-cellular elements part of the extracellular brain microenvironment (Volterra et al., 2014; Ribot et al., 2021; Semyanov and Verkhratsky, 2021). Further investigation is needed to elucidate the emerging complexity of mechanisms and dynamics mediating specific types of astrocytic Ca^2+^ patterns and astrocyte processing of information.

## Calcium and gliotransmitter release from astrocytes

**FIGURE 2:**
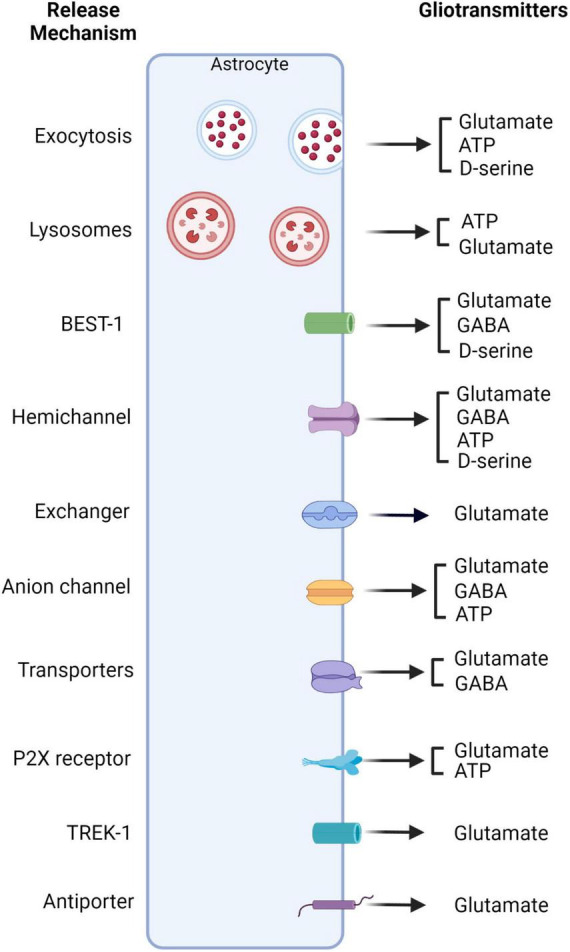
Schematic of Ca^2+^ dependent and independent gliotransmitter release. Astrocytes can release gliotransmitters through a variety of mechanisms dependent and independent of calcium. Glutamate has been shown to be released *via* a variety of mechanisms. These mechanisms include exocytosis, lysosomes, hemichannels, exchangers, anion channels, antiporters as well as channels such as TREK-1 and Bestropin-1 (BEST-1) (Araque et al., 2000; Montana et al., 2004; Zhang et al., 2004; Xu et al., 2007; Malarkey and Parpura, 2008; Yang et al., 2019; Okada et al., 2021). GABA on the other hand, has been shown to be released *via* BEST-1, hemichannels, as well as anion channels and transporters (Kozlov et al., 2006; Jiménez-González et al., 2011; Le Meur et al., 2012; Yoon and Lee, 2014; Christensen et al., 2018; Kwak et al., 2020). ATP can be released *via* hemichannels, exocytosis, anion channels, and lysosomes (Bezzi and Volterra, 2001; Fujii et al., 2017; Xiong et al., 2018). Lastly, D-serine has been shown to be released *via* exocytosis, BEST-1, and hemichannels (Wolosker et al., 1999; Martineau et al., 2013; Sild and Van Horn, 2013; Herman, 2018; Koh et al., 2022; Linsambarth et al., 2022; Park et al., 2022; Tapanes et al., 2022). Created with BioRender.com.

Since the coining of the term, “tripartite synapse,” researchers have been studying the extent that astrocytes actively communicate with neurons (Araque et al., 1998, 2014). One of the active mechanisms of astrocytes that impacts synaptic transmission is gliotransmission (Araque et al., 2014). Gliotransmission refers to the capacity of astrocytes to release neuroactive molecules that impact synaptic transmission or neuronal signaling (Araque et al., 2014; Volterra et al., 2014). Many of these signaling molecules include classic transmitters such as glutamate and GABA and amino acids like ATP/adenosine and d-serine. Even though the cellular and molecular mechanisms mediating gliotransmitter release are not completely understood, several studies have revealed both calcium-dependent and -independent release mechanisms (Guček et al., 2012; Li et al., 2013; Sloan and Barres, 2014; Figure 2).

### Glutamate

Calcium-dependent and -independent mechanisms for glutamate release from astrocytes have been proposed. These include (a) exocytosis from vesicles, (b) anion channel opening, (c) glutamate exchange *via* cystine-glutamate antiporter, (d) release from hemichannels, or (e) ionotropic purinergic receptors (Araque et al., 2000; Montana et al., 2004; Zhang et al., 2004; Malarkey and Parpura, 2008). Vesicular gliotransmitter release of glutamate has been supported by morphological and functional evidence. For example, it has been shown that astrocytes possess some of the proteins involved in exocytosis, including the soluble N-ethyl maleimide-sensitive fusion protein attachment protein receptor (SNARE) complex (Zhang et al., 2004), to control vesicle fusion. SNARE proteins, such as VAMP2 or VAMP3, Syntaxin 1, SNAP23, and synaptotagmin isoforms have been detected in astrocytes (Bohmbach et al., 2018; Mielnicka and Michaluk, 2021). Interestingly, the mechanisms involved in glutamate-mediated exocytosis have been highly debated in the last years (Li et al., 2008; Chai et al., 2017). Functionally, expression in astrocytes with the light chain of tetanus toxin that selectively cleaves the vesicle-associated SNARE protein potently inhibits the release of glutamate from astrocytes (Montana et al., 2004; Xu et al., 2007; Araque et al., 2014). However, complementary evidence has questioned the exact mechanisms involved in Ca^2+^-dependent glutamate exocytosis (Li et al., 2008; Chai et al., 2017). Deployment of a variety of experimental approaches revealed that fusion events from astrocytic vesicles following intracellular calcium increase occurs in a much slower time scale in comparison to neurons (Bezzi et al., 2004; Calì et al., 2008; Marchaland et al., 2008). While in neurons the fusion occurs in less than 0.5 ms following calcium increase, in astrocytes the exocytotic release takes place over two orders of magnitude slower (Bezzi et al., 2004; Calì et al., 2008; Marchaland et al., 2008; Südhof, 2012). The release of glutamate may also occur *via* the opening of glutamate-permeable, two-pore domain potassium channel TREK-1 or the opening of glutamate-permeable, calcium-activated bestrophin anion channel (Best1). Ultrastructural analyses demonstrate that TREK-1 is preferentially localized at cell body and processes, whereas Best1 is mostly found in microdomains of astrocytes near synapses (Woo et al., 2012). Recent evidence has also shown that activation of volume-regulated anion channels (VRAC) can lead to glutamate release. When this channel is activated by cell swelling, astrocytes in the hippocampus release glutamate (Yang et al., 2019). Lastly, glutamate may also be released *via* hemichannels which can be blocked by drugs targeting synaptic vesicle protein 2A (Okada et al., 2021).

### GABA

GABA is an important neurotransmitter for neuronal inhibition. As neurons, astrocytes can also release GABA *via* transporters, anion channels, and gap junction channels (Yoon and Lee, 2014). In contrast to glutamate, GABA release from astrocytes has been reported to be mediated by distinct mechanisms, as the vesicular release of GABA seems unlikely, due to the lack of GABA-containing vesicles in astrocytes. Atypically, astrocytes synthesize GABA from the polyamine putrescine using monoamine oxidase B (Yoon and Lee, 2014). Early examples of GABA release from astrocytes have been found in the olfactory bulb, thalamus, and hippocampus (Kozlov et al., 2006; Jiménez-González et al., 2011; Le Meur et al., 2012). One of the major functional consequences of astrocyte-derived GABA is the tonic inhibition of various neuronal circuits. Various mechanisms of GABA release from astrocytes have been proposed. Calcium-dependent GABA release from astrocytes potentially involving the GABA transporter GAT has been reported in the dorsal root ganglia (Christensen et al., 2018). Other mechanisms for GABA release from astrocytes such as Best anion channels and gap Junction hemichannels have also been described. “Sniffer-patch” experiments have shown that the Best-1-mediated release of GABA is dependent on intracellular calcium and is triggered by GPCR activation. Tonic inhibition caused by GABA release *via* glial Best1 anion channels has been reported in the cerebellum and thalamus (Lee et al., 2010; Kwak et al., 2020). This mechanism has also been demonstrated in reactive astrocytes in the hippocampus (Pandit et al., 2020). Finally, gap junction hemichannels could be another route by which GABA can be released from astrocytes. GABA release *via* gap junction hemichannels is involved in the regulation of tonic GABA currents of neurons in cultured hippocampal neurons and acute hippocampal slices (Ransom et al., 2017).

### ATP

ATP is a primary energy source in cells and also acts as an important messenger molecule through action on purinergic receptors. ATP plays an important role in calcium wave propagation in astrocytes (Bezzi and Volterra, 2001). Unlike the previously mentioned gliotransmitters, the mechanism for exocytosis was unclear *in situ* until recent years. This was due primarily to using indirect assays to measure quantal and non-quantal ATP release (Xiong et al., 2018). Many studies have examined calcium-dependent and independent mechanisms of ATP release. Evidence collected from mice conditionally expressing the SNARE domain of VAMP2 selectively in astrocytes (dn-SNARE mice), has shown Ca^2+^-dependent ATP release by astrocytes (Lalo et al., 2014). In addition, ATP release can be mediated by calcium-dependent lysosome exocytosis (Pangršič et al., 2007; Zhang et al., 2007). Lysosome exocytosis and ATP release occurred after mechanical stimulation in primary hippocampal astrocyte culture (Xiong et al., 2018) in a calcium-dependent manner (Lee et al., 2015). In addition, ATP can also be released *via* connexin 43 (Cx43) hemichannels and anion channels (Kang et al., 2008; Fujii et al., 2017).

### D-serine

Astrocytes can produce and store D-serine in vesicles (Martineau et al., 2013; Sild and Van Horn, 2013). The enzyme, serine racemase converts L-serine to D-serine (Wolosker et al., 1999). Astrocytes play an important role in the serine shuttle by converting L-serine from glucose which can then supply to neurons (Herman, 2018). Ca^2+^ dependent vesicle release of D-serine has been demonstrated to modulate long-term potentiation (LTP) (Henneberger et al., 2010; Bergersen et al., 2012). Astrocytic glutamate activates on mGluRs and further activates LTP in cholinergic neurons (Navarrete et al., 2012). Moreover, astrocyte release of D-serine also leads to LTP modulating recognition memory (Robin et al., 2018). Glial D-serine is relevant for astrocytes across multiple species including Drosophila. In Drosophila, glial D-serine is required for thirst-directed behavior (Park et al., 2022). Many studies have shown that astrocytes can release D-serine under pathological conditions. For instance, preventing the release of d-serine from glia reduce synaptic damage after traumatic brain injury (Tapanes et al., 2022). Astroglial d-serine can also travel through Cx43 hemichannels. The form of release is particularly important for fear memories during fear conditioning. Blocking Cx43 in the basolateral amygdala impaired fear memory consolidation (Linsambarth et al., 2022). In addition, astrocytes can also release D-serine *via* Best1 channels. This has been shown to alter NMDA tone in the hippocampus (Koh et al., 2022).

## Conclusion

The development of tools for visualization and manipulation of cell Ca^2+^ dynamics together with advances in imaging techniques have enabled the monitoring and modulation of astrocyte Ca^2+^ signaling in *in vitro*, *ex vivo*, and *in vivo* preparations (Li et al., 2013). Advanced optical imaging techniques, sensitive genetically encoded Ca^2+^ indicators (GECIs), and optogenetic and pharmacogenetic tools allow the selective measuring and activation of astrocyte Ca^2+^ signaling pathways to study astrocyte-neuron communication, mechanisms of gliotransmitter release, and role of astrocytes in physiology (Li et al., 2013; Semyanov et al., 2020). In particular, selective astrocyte GPCR activation has been useful to explore the functional role of astrocyte Ca^2+^ signaling in specific brain areas and astrocyte populations (Losi et al., 2017). A variety of experimental approaches are now available to increase astrocyte intracellular Ca^2+^ levels, such as light-gated glutamate receptor, channelrhodopsin-based effectors, melanopsin, optoXRs, and designer receptor exclusively activated by designer drugs (DREADDs) (Hirbec et al., 2020). In particular, Gq-GPCR and Gi-GPCR DREADDs have been widely used in the field, as they offer an opportunity for non-invasive and selective *in vivo* activation of astrocyte GPCR pathways after selective agonist administration (Losi et al., 2017). Even though there is a variety of tools to increase astrocyte Ca^2+^ signaling, till recently, IP_3_R2^–/–^ mice and IP3 sponges (Agulhon et al., 2008; Petravicz et al., 2008) have been the only available options to achieve astrocyte Ca^2+^ selective attenuation. Recent studies have provided new tools to lessen intracellular Ca^2+^ release, such as activation of kappa-opioid receptor coupled to a Gi-GPCR selectively activated by salvinorin B (Vardy et al., 2015; Herrera Moro Chao et al., 2022) or by Cre-dependent expression of hPMCA2, a human plasma membrane Ca^2+^ ATPase pump that constitutively extrudes Ca^2+^ from astrocytes (Yu et al., 2018, 2021). Decreases in astrocyte intracellular Ca^2+^ levels have also been observed during neuropathology after astrocyte Gs-GPCR activation (Pham et al., 2021).

The evolving genetically targeted optical and pharmacological tools to modulate astrocytic Ca^2+^ signals have been of value in several studies in the field, showing that astrocyte function and astrocyte-neuron communication is heavily impacted during pathological conditions (Nedergaard et al., 2010; Nanclares et al., 2021; Herrera Moro Chao et al., 2022). Visualization of astrocyte Ca^2+^ by GECIs monitoring has shown that astrocytes become hyperactive in many neurological diseases such as traumatic brain injury, amyotrophic lateral sclerosis, epilepsy, and Alzheimer’s disease (AD) (Shigetomi et al., 2019). In addition, modified gliotransmitter release and synaptic transmission have been associated with the development of astrocyte hyperactivity and reactivity (Nedergaard et al., 2010; Nanclares et al., 2021; Herrera Moro Chao et al., 2022). In conclusion, further studies are essential for a precise understanding of the detailed mechanisms by which astrocyte-neuron communication mediates physiological outputs and how the dysregulation of this reciprocal communication affects the development of neuropathology. Tailoring novel molecular tools that specifically modulate astrocyte Ca^2+^ signaling pathways combined with advanced Ca^2+^ imaging techniques *in vivo* will further shed light on the complexity of astrocyte-neuron bidirectional communication and its impact on physiology.

## Author contributions

JG, AA, PK, and DH drafted manuscript and approved the final version of the manuscript. All authors contributed to the article and approved the submitted version.
